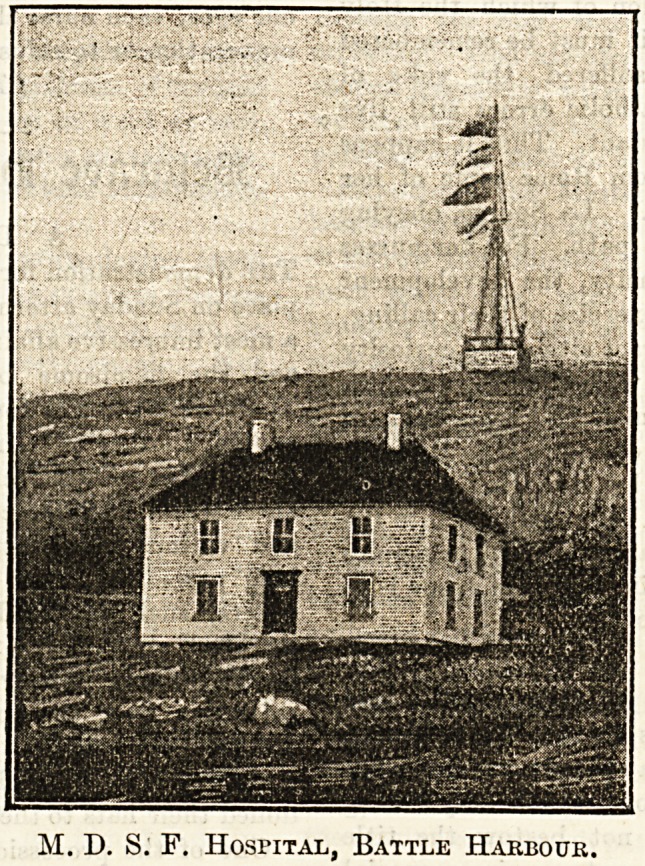# Extra Supplement.—The Nursing Mirror

**Published:** 1893-11-11

**Authors:** 


### "The Hospital" Nursing Mirror.

Being the Extra Nursing Supplement of "The Hospital" Newspaper.

[Contributions for this Supplement should be addressed to the Editor, The Hospital, 428, Strand, London, W.C., and should have the word "Nursing" plainly written in left-hand top corner of the envelope.]

### News from the Nursing World.

### OUR CHRISTMAS COMPETITIONS.

We anticipate that an immense variety of garments will this year be made by our readers for the adult sick poor who have to spend their Christmas Day within hospital walls. The parcels which are the result of our annual competition receive such a hearty welcome at each institution to which they are sent, that we hope to see them increase in bulk and in number every year. The prizes will be: (1) For the best pair of socks knitted by a nurse, 5s.; (2) for the best pair knitted by any Hospital reader, 5s.; (3) for the best made flannel shirt, 10s.; (4) for the best flannel or flannelette bed-jacket, 10s.; (5) for the best flannel petticoat, 10s.; (6) for the best made and simplest shaped dressing gown made and cut out by a nurse, 20s. Long seams may be done by machine. With the view of enabling an estimate to be formed of the progress made, we shall be glad to receive the names of competitors, and to hear which of the six prizes each will enter for. All parcels must reach The Hospital Office, 428, Strand, in the first week of December, and they should bear the words "Needlework Competition" in left-hand corner of address label, and also the name of the sender.

### UNWELCOMED FRANKNESS.

"They didn't thank me for telling them!" is the remark frequently uttered by some honest body who has given a truthful but unwelcome answer to questions officially asked. Recently, when "bad throats" prevailed in a large household, defective drainage being subsequently discovered, "I pointed out the suspicious spot," said one who was living there, "and the inspector detected the evil at once, but no one thanked me for bringing expense on the establishment." Apparently the officials did not recognise any merit in the person who probably saved them from the grievous consequences of an epidemic much more serious than these premonitory "suspicious throats." Another person who gets no thanks, is the nurse who tells the truth about diets. A question as to the quality of the patients' food is easily answered, "Bread good, milk good, potatoes bad," but moral courage begins to evaporate when nurse learns that the committee gentleman who is "going round" considers "there's nothing the matter that I can see! I wish the potatoes they give me at home were half as good." Nine times out of ten the nurse-critic ends by feeling that she must be a grumbling and fault-finding woman, and that she holds perverted ideas regarding the cooking and quality of vegetables. If reasonable complaints of food or of drains were patiently received, those who are wise in their generation would help forward the reforms which at present they dare not attempt because "no one will thank them" for giving their true impressions and experiences.

### HOSPITAL AND ASYLUM WORKERS.

The second volume of "The Census of England and Wales, 1891," recently issued, gives the inmates in general, special, convalescent, and sanitary hospitals, including officers and their families, as 40,341, of which 15,607 were males and 24,734 were females. The figures in the census show that the officers and their families represented a total of 12,762 persons, of whom 1,545 were males and 11,217 were females. Deducting the number of patients, these figures show that there was one male or female officer to every two and one-thirteenth patients. Of the 11,217 women workers in hospitals, we estimate that 9,000 were nurses and 2,124 represent ward maids and other inmates than patients. It must be borne in mind that these 9,000 nurses are exclusive of those to be met with in workhouse infirmaries and lunatic asylums, as well as of the majority of those engaged in private nursing. The workers in public lunatic asylums numbered 8,601, of whom 3,109 were males and 5,492 were females. These figures probably represent one female attendant to every six patients, and one male attendant or officer to every nine male patients. Of the 5,492 female workers, 3,551 appeared to be nurses or attendants in charge of the inmates. It will be seen from these figures that the cost of attendance and service for the sick is from two to three times as great in a hospital as it is in a lunatic asylum, a fact which should be borne in mind by those who may wish to compare the relative cost of hospitals and lunatic asylums.

### HEAVY CASES.

To define a "heavy case" is by no means easy. Just as one person considers he has had a busy day after merely accomplishing as much work as a more experienced or more industrious man would get through in a couple of hours, so one nurse talks glibly of a case as "heavy" which her wiser contemporary would look upon as quite ordinary. Generally speaking, "heavy" may be taken to mean an exceptionally severe case of illness which puts an abnormal strain on the attendant. But who is to be the judge? An unselfish invalid is morbidly conscious of the trouble he gives, whilst a selfish one accepts everything as his right. Patients' friends are naturally prejudiced on one side, and the nurse has her own views on the other; and the doctor, again, is generally willing to advise the engagement of a second nurse when necessary, if he be consulted. It is not merely in paralysis nor in acute illnesses that the word "heavy" is universally adopted by the indolent or avaricious nurse who demands higher fees on account of the "trying nature of the case;" yet, naturally, private families seldom burden themselves with the presence and expense of a stranger until the patient is beyond their own skill. When they engage a trained nurse they certainly have a right to her services; there is no favour on either side in a strictly business arrangement. A little patience and mutual accommodation should obviate all need for any single ordinary case of illness being called "heavy." When a nurse demands double pay because she has done double work, the claim reveals a wrong state of things. The patient must have inevitably suffered from an arrangement by which one overworked person attempted duties which could only be properly performed by two competent attendants.

### THE CIVIL HOSPITAL, ST. HELENA.

Miss R. A. Blennarhasset andl Miss Sleeman having rapidly completed their book, "Adventures in Mashonaland by Two Hospital Nurses," which will be published by Macmillan in a few days, are not going to rest on their oars. Miss Blennarhasset has been selected out of a large number of applicants for the post of Superintendent of the above hospital, and Miss Sleeman, whose health has been seriously tried in Mashonaland, is going with her, but not to work. The climate of St. Helena is said to be delightful, and we wish "the two hospital nurses" God-speed and much prosperity and happiness in their new field of duty.

### ANOTHER NEW HOME.

The new Nurses' Home at the Blackburn Infirmary, opened by Mrs. Henry Harrison, of Stanley, on October 30th, is a handsome and commodious building containing fifty rooms. The nurses must rejoice in the new quarters, which have superseded the very inadequate accommodation which has hitherto served for them. One of the speakers at the opening ceremony suggested the addition of some district nurses to attend the sick poor in their own homes. It is to be hoped that this plan may prove possible.

### A PRAISEWORTHY DECISION.

At Melbourne a number of women disguised as nurses have made themselves notorious by selling patent medicines and other wares from house to house. Their uniform obtained for them admission into dwellings where they would not have been allowed to enter had their calling been suspected. Many complaints having been addressed to the police on the subject, Mr. Panton, P.M., "expressed the opinion that licences should not be granted to women who practised the deceit of employing a nurse's costume as a cloak to the real object of their business." As a result of this intimation, many women who were in attendance withdrew from the court without applying for the hawker's licences, which they had evidently come in search of.

### A TORONTO ASSOCIATION.

The "Nursing at Home" Association at Toronto has now a conveniently arranged dispensary in connection with it. Here burns and all kinds of wounds are dressed daily with great skill and care, prescriptions are made up, the small fees charged for the medicines going towards the working expenses. The leading doctors of the city attend for an hour at a time. The dispensary is intended entirely for the destitute poor, unable otherwise to secure the advantage of medical treatment. Various friends of the "Nursing at Home" Association have given most useful gifts during the summer months, and these have considerably lessened the expenditure. Funds for the maintenance of the association are obtained from private donations, and by a grant from the city, and the accounts at the last meeting showed a balance of two dollars of income over expenditure. The dispensary appears to be a most valuable addition to the resources of this useful association.

### VANISHED JOYS.

There is sadness amongst the Nursing Sisters in India because, by recent regulations, they are forbidden to go to balls, theatres, and concerts. Race meetings and afternoon parties are still countenanced, and the keep of the nurses' horses, when they are on active service, has also been lately arranged for. But these things do not console lovers of valses and polkas, and they now feel themselves very hardly dealt with. We have ascertained that copies of the new order are not yet obtainable in England, so we must withhold judgment until all the facts are known. It remains to be seen if any and what modifications are desirable in the true interests of the best nurses. A modification of these regulations to permit the Nursing Sisters to attend theatrical performances may perhaps be hoped for. There is, of course, no reason against nurses dancing whenever they wish it, but their own active work seldom leaves them with much inclination for violent exercise of any kind except, perhaps, during their annual holidays.

### SHORT ITEMS.

An inquest was held the other day at Hampstead on the body of a woman who had died in the workhouse. Complaints having been made by the friends as to the treatment which the deceased had received from a night attendant, the jury appended this rider to their verdict "only properly-trained nurses should be employed to take care of the sick and aged in the workhouses."—Additional and improved accommodation for the nurses at Derby Workhouse is under discussion.—A new Nurses' Home has just been opened at Whitford Lodge to accommodate members of the Ockley Association.—The Reading Guardians have acceded to the wishes of their workhouse nurses, and have granted them indoor uniform.—The new Home of the Leeds District Nursing Association in now completed, and is suitable for the requirements of the four or five nurses whom it is destined to accommodate. Small branch Homes are also doing good work in crowded districts.—The Guardians have referred the proposed inquiry into the nurses' grievances at Islington Infirmary to the consideration of the House Committee.—The North Dublin Union have appointed Nurse Edith Jackson as assistant night nurse in the Protestant wards.—The show of chrysanthemums at the Royal Aquarium was a most beautiful one, and the enormous size and great variety of the blooms exhibited show the attention which is now paid to this branch of horticulture.—The Church Army Nurses are expected to secure the St. John Ambulance certificate for "First Aid," and they attend nursing lectures; they also spend a few hours daily for six weeks at Kensington Infirmary, but the Hon. Superintendent states that they are not sent out as trained nurses.—Miss Annie Morgan, of the N.H.S., has just completed a successful course of Homely Talks on Health and Ambulance, at the various centres of the Torrington district, under the Devon County Council. The lectures were well attended, and at the last Miss Morgan was presented by her audience with a collection of views of the neighbourhood.

### On the Nursing of Diseases of the Stomach.

### I.—INTRODUCTORY.

Before considering the management of certain diseases of the stomach, it will be well to consider briefly some points in the anatomy and physiology of that organ. The stomach is the widest part of the alimentary canal; it is a bag with two openings in it, one leading from the œsophagus, and this from its position near the heart is called the cardiac orifice, and the other opening, named the pyloric orifice, leading into the duodenum, or first part of the small intestine. On the left side of the cardiac orifice the stomach bulges upwards and backward, and this bulging part is called the great cul-de-sac or fundus. Between the two openings are two curved borders; the upper one, the lesser curvature, is short and concave; the lower one is much longer and convex, outlining the fundus, and is the greater curvature.

The size of the stomach varies with the amount of distension. When moderately filled, it is about twelve inches long and four to five inches across at its widest part. In the adult, it is capable of holding about six to eight pints of water; in the infant at birth it can hold rather less than an ounce and a half, but at a month old it is capable of containing more than four times as much as at birth, and at two years old it takes a pint and a half.

Next as to the position of the stomach. It is situated at the upper part of the abdomen, and when empty lies at the back of that cavity and beneath the liver, but when distended it is brought forwards, so as to lie immediately behind the abdominal wall. Thus, it is more liable to be injured when full than when empty. But the stomach also extends upwards behind the lower ribs on the left side, and the fundus is a little above and behind the spot where the apex beat of the heart is felt, being separated from the heart and base of the left lung by the diaphragm. Thus the stomach occupies a part which is very commonly regarded as belonging to the thorax. From this, it can easily be understood how important it is not to overload the stomach in cases where the heart or lungs are diseased. For example, in cases of chronic bronchitis and emphysema a distended stomach pushing upwards the diaphragm, and thus diminishing the space available for the lungs, much increases the difficulty of breathing. Hence the need for light and easily digested food in such cases. On the other hand, it is very common for a patient to complain of his heart, when it is the function of the stomach which is primarily at fault. Pains in the stomach are supposed by the patient to have their seat in the heart; or over-distension of the stomach may give rise to palpitation, the heart itself being sound.

Next as to the function of the stomach. The stomach receives through its cardiac orifice food which has been masticated, mixed with saliva, and swallowed in larger or smaller masses. It discharges through the pyloric orifice semi-fluid chyme. The changes in the food, during its stay in the stomach, are brought about mainly by two factors: (1) the movements of the stomach; (2) the action of the gastric juice. The movements of the stomach are of two kinds. First the churning movement by which the food is moved along the great curvature and back along the small curvature, so that it may be fully exposed to the action of the gastric juice. Secondly, the propulsive movement, which begins when digestion has been going on for a short time, and by which the food is driven through the pylorus into the duodenum. But if large masses of undigested food are driven against the pyloric orifice, the opening is closed against them, and they are carried back into the main part of the stomach to be again acted upon by the gastric juice. These movements are brought about by the contraction of the muscular coats of the stomach, the contraction being usually called forth by the presence of food. Muscular contraction of the coats of the stomach is regulated by, but not dependent upon, nerves which supply the stomach. There are two sets of nerves, one set tending to increase the movements, the other set to diminish them.

The gastric juice is secreted by glands of the mucous membrane of the stomach. The secretion is poured out when food enters the stomach, and this is the most effective stimulus. But the flow is, to a certain extent, influenced by nervous action; for instance, the appearance of food may excite a flow of gastric juice; emotions, such as fright or fear, may arrest the flow. Gastric juice is a thin, almost colourless, fluid, containing about ·2 per cent. of hydrochloric, acid in which medium the ferment pepsin is able to act upon nitrogenous food, changing it into a more soluble form. Neither fats nor carbohydrates (such as starch and sugar) are digested by gastric juice.

The blood supply of the stomach will be considered in treating of hæmatemesis, but it may be remarked here that of course both the movements and secretion of the gastric juice are dependent on a healthy circulation in the walls of the stomach.

*Hiccough* is caused by a sudden descending jerk of the diaphragm. The descent of the diaphragm makes the cavity of the chest larger and, therefore air enters into the lungs through the glottis, or upper opening of the larynx. But the diaphragm is acting out of its proper time, and so the glottis is not properly open, and the air going through the narrowed opening makes the well-known noise. Hiccough is generally caused by the presence of undigested food in the stomach. This irritates the nerves of the stomach and reflexly causes the diaphragm to contract. Or it may be produced by direct irritation of the diaphragm, as by inflammation of the peritoneum on the under surface of the diaphragm. Hiccough is generally a trivial ailment, but may be serious if it occur with great persistency in the course of an exhausting illness. Occasionally hiccough occurs apart from disturbance in the abdomen as a neurosis. Thus, in hysterical cases it may be very loud and very constant. The treatment of hiccough will depend upon the cause. If dependent upon gastric disturbance, the digestion must be attended to. But apart from treating it in this way, nurses might be called upon to deal with an attack, and then besides the well-known remedy of holding the breath for as long as possible, a teaspoonful of chloroform water might be ordered. A mustard leaf, or a warm application of some sort, to the epigastrium affords relief in other cases.

### Where to Go.

Royal Albert Hall.—Free organ recitals every Sunday afternoon at half-past three.

Working Men's College, Great Ormond-street. — Free Lecture by Rev. Boyd Carpenter at half-past eight on Saturday, November 11th.

Mission to Deep Sea Fishermen.—Public meeting in Exeter Lower Hall on Thursday, November 30th, at seven p.m.

North London Nursing Association.—A sale of work will be held at the Home, 413, Holloway Road, on December 7th and 8th.

Brixton Hall.—A monster Christmas tree will be exhibited at Brixton Hall on Tuesday and Wednesday, December 19th and 20th, in connection with the Brixton Orphanage, Barrington Road, S.E.

### Nursing in the United States.

(By Miss Darche, Lady Superintendent New York Training School for Nurses, Blackwall Island.)

### PROPER ORGANISATION OF TRAINING SCHOOLS.

(*Continued.*)

The school which had thus far been developed as nearly as had seemed practicable on the plan for training nurses established by Florence Nightingale at St. Thomas's Hospital, London, England, now began to assume proportions and a bearing distinctly American. The school had been started on very democratic principles; the women who entered to train, whatever their previous condition of life, were placed on the same level and plane, and none were engaged to do the higher or more special or nicer parts of the hospital nursing. They all began at the bottom, to work on and up to the more responsible duties, as they showed capacity for advancement.

At first, of necessity, the undergraduates were obliged to act as head nurses of the wards, the importation of head nurses or "sisters" from abroad being too expensive to be contemplated, and, as yet, there were no graduate nurses here. But, by degrees, what was at first regarded as a misfortune, came to be considered a part of the system, and it was found that by extending the course of training from one year to two, the services of the nurses, after they had obtained the practical training of the first year, could be retained and utilised as head nurses. Had they graduated at the end of the first year, and been at liberty to withdraw from the hospital, so great had the demand become for their services outside, that no salary the hospital or school authorities could afford to offer would have been sufficient to retain them even as head nurses in the hospital for a second year. Aside from this view of the case, it soon became apparent that the second year of hospital service proved to be very valuable to the nurse herself, training her in self-reliance, perfecting her in technique, developing her judgment, and giving her that power to control others, which is a very essential quality in the make-up of a nurse. During the second year also, nurses at first were very generally sent out to nurse in private families, and in this way to add to the funds of the school; but of late years this plan of utilizing the services of a nurse during her second year has been very generally discarded by our larger and better established training schools. In this way was inaugurated the "two years' course of training," now so generally adopted and advertised in connection with our American training schools.

At first sight it would appear that the course of training so called, had in this country been extended over a longer period of time than the training period of the nurse at St. Thomas's Hospital, London, England; but on investigating the subject closely, and on all sides, we find that this is not the case. To quote from the St. Thomas's circular of information:—

"The Committee of the Nightingale Fund have made arrangements with the authorities of the St. Thomas's Hospital for giving a year's training to women desirous of working as hospital nurses. At close of year their training will usually be considered complete, and during the three years next succeeding the completion of their training, they will be required to enter into service as hospital nurses in such situations as may from time to time be offered them by the Committee. At the end of a year those whom the Committee find to have passed satisfactorily through the course of instruction and training will be entered on the register as certificated nurses, and will be recommended for employment accordingly. The Committee have hitherto readily found employment for their certificated nurses in some public hospital or infirmary at salaries beginning at £20, with board, washing, &c. Engagements are not to be made except through the Committee, and no engagement must be terminated without a quarter's notice to the Committee. The training establishment is maintained by the Nightingale Fund, which was established, &c., &c. The nurses so trained are drafted into other public institutions, but it is not the object of the Fund to train for private nursing. We do not give our nurses printed certificates, but simply enter the names of all certificated nurses in the register as such.

"*Obligations signed by probationers after one month's trial*: 'Having now become practically acquainted with the duties required of a hospital nurse, I am satisfied that I shall be able and willing on the completion of my year's training to enter into service for the space of three years at least in whatever situation the Committee shall think suitable to my abilities, it being my intention from henceforth to devote myself to hospital employment, and further agree not to enter into any engagement without first having obtained the approval of the Committee, and not to leave any situation without giving due notice to the Committee.

Signed ———.'"

From the above it would appear that a woman wishing to train as nurse at St. Thomas's must, after passing satisfactorily a month of trial, sign a paper promising to give four years of service under the control and management of the Nightingale Fund Committee, the first year as probationer under training, the other three to act in the capacity of a hospital nurse. It would also appear that although at the end of the first year she is duly registered as a certificated nurse in the hospital books, she is not given her certificate, nor even after the fourth year does she receive it.

From these facts it would seem that a nurse is never graduated in the sense of being placed on her own responsibility, but must always remain under the supervising control and authority of the training committee. It is not surprising, then, that a departure from the original system should have been found necessary in this country, where the idea of pledging oneself to a corporation or society for a period of four years, not to say indefinitely, would have been regarded as almost impossible. The Board of Managers of the first Training School in America, therefore, very wisely decided that a compromise between the one year thought necessary for training, and the four years pledged by the St. Thomas's system, and offered a two years' course. They also decided at the end of the two years, the nurse having proved worthy, should receive the diploma or certificate of the school, with liberty to choose for herself her future line of action; and they further decided upon a yearly commencement, where the members of the graduating class each year might receive with a parting address, the public and honourable recognition of the close of their service as nurses of the school.

Nor, again, is this departure from its original model to be wondered at, when we remember that the aim of the Nightingale Fund, connected with St. Thomas's Hospital, was, first, to train women as nurses, and then to draft them off to other hospitals and infirmaries, where pledged to a central committee of control, they worked out the chief purpose of the fund in reorganising and reforming the hospitals and infirmaries in which they were placed. The purely philanthropic spirit of this "missionising and reformative" plan has not only accomplished the wise purpose of its founder in revolutionizing the nursing in hospitals over all the English speaking world, and that in an incredibly short space of time, but it has gone further, and through secondary channels has provided skilled nursing in large measure for the sick wherever placed, whether rich or poor. It has also provided a useful, honourable, and self-supporting work for many women.

(*To be continued.*)

### Wants and Workers.

Can anyone recommend an institution where a man, aged 30, deaf and dumb, can be received and taught to earn his own living? His friends can pay a little towards his maintenance. The man has received some education, and belongs to a respectable family. Particulars can be obtained from E. P., Cottage Hospital, Legburthwaite, Grasmere.

### Nursing in Newfoundland.

(By Our Own Correspondent.)

### MISSION TO DEEP SEA FISHERMEN.—LABRADOR.

(*Continued.*)

We were often summoned long distances to see sick people. Once we went five or six miles in a boat through a fog, and when we thought we had nearly arrived at our destination, we found in front of us a long bar of rocks stretching far out to sea. The boat had to be turned round quite suddenly, and we were nearly upset. The men took down the sail as quickly as possible, and then found that an oar had been lost. Fortunately two still remained to us, for we had two miles further to go.

It was not a pleasant experience, although we did not feel any fear. Afterwards we had a long walk over rocks and through bogs, and visited all the huts in the place. On an average five men and two girls inhabited one sleeping room, and sometimes four men and one girl, and there is much immorality. We could not get back to the "Albert," so we were obliged to stay for the night in the largest of the houses.

The evening service held daily on board was always well attended; in fact, we hardly knew how to bestow the people, and on Sundays we had three services.

The patients were always attended to after the services, and we had long busy evenings. On arriving at each new place we were immediately besieged by cases of poisoned fingers and hands, phthisis, heart disease, anæmia, night-blindness, sarcoma, and a great deal of constipation. Then there were the patients to visit on shore, and to distribute nourishment to some of them, their ordinary diet being bread, fish, tea, molasses, and no vegetables.

M. D. S. F. Hospital, Battle Harbour.

Indian Harbour, where it is proposed to build our most northern hospital, is about 150 miles from Battle Harbour, and an even more barren-looking place. At first we could only see two white houses, but afterwards discovered various huts dotted about on the hillside, and of so much the same colour that it was difficult to distinguish them.

The wood of which our future hospital is to be built had only just been landed on the rocks, but the men soon set to work with it. Many sick persons were brought to us at Indian Harbour from miles round "to see the doctor."

We visited a man in a "bunk-house" very ill with gastric ulcer. It was a wretched place, containing three bunks a yard in width, two men sleeping in each, with their heads at opposite ends. The door was very old, and such as might serve for an outhouse at home, and it did not reach the lintel by some inches. The cold wind entered freely, and the room was very damp. We paid many visits to this patient, and used to sit and read to him. We also looked after two sick girls.

We were on one occasion summoned to a sick person at a distance, and went in the boat sent for us, taking medicine and clothes for the patient and provisions for ourselves. We found the household consisted of eight half-bred Eskimos living in one room. Going a little farther we put up at a house, where we had some tea, and invited the family to join us. They were terribly dirty! Afterwards we went on to a very wretched hut, in which lived a man and his wife and two children, the man very bad with phthisis; the bedding consisted of rags, and he was very poorly clad. For food they had only a little broken bread, *i.e.*, hard biscuits. They had no shot nor powder, and no proper fishing gear. They had caught no fish for a day or two, and did very badly last winter. Shot and powder were supplied to them shortly after by the mission. We had a short service at our lodging, and I slept on the floor of a loft on some of the clothes we had brought with us. My ulster rolled up served as a pillow, and a rug represented bedclothes. The wind was very high during the night, and combined with the mosquitoes to make sleeping difficult. Next day we distributed clothing and medicine. In returning we had to go in three different boats, and at one stopping-place we had herrings and tea for our dinner, and reached the "Albert," just at the time of evening service, having seen seventeen patients. We were called out to see a woman who had been taken ill one very beautiful night, when the sea was full of phosphor essence, and we did not get back till after eleven, and there came another summons for "the doctor" after that.

One day we were sent for to see a poor woman who had been terribly hurt by the roof of a fish-stage falling upon her, and we went off in a boat on a very rough sea. Then we walked across Indian Island, and on by boat to another island, returning by the light of a lantern. Dr. Grenfell returned to us in the "Princess May," having been down the Straits of Belle Isle preaching, and also visiting many sick persons.

Finding the hospital at Indian Harbour could not be finished this year, he endeavoured to get a house or store to fit up as a temporary hospital, but none could be found. This was a great disappointment, as we had hoped to do much for the poor people here. It was therefore decided to return to Battle Harbour and help forward the work there.

Leaving the "Albert" at Cape Harrison, a small steamer carried us the rest of the way. A great deal of good work has been done at Battle Harbour, where eight beds and a cot have been kept constantly occupied. Two patients have died, one from phthisis, the other from scurvy; and we have nursed poisoned hands, ruptured perineum, hip disease, abscesses, gun-shot wounds, &c. Four patients for whom we had no room have been placed in lodgings and nursed there. A steward does the cooking in the hospital, and a young girl manages the housework. Patients around here are just like English ones, but further up north there are many Eskimo and half-breeds.

It must be borne in mind that the "Albert" is the first mission ship sent out to Labrador, and that there are 8,000 permanent residents and about 25,000 summer visitors entirely cut off from medical aid. The value of the medical work of the M.D.S.F. is therefore incalculable, and many more workers could find ample employment.

This mission sends out only fully qualified medical men, and this year, for the first time, two trained nurses have also been secured.

Warm clothes of all descriptions are constantly required, and donations or subscriptions are gladly received by the Secretary at the Bridge House, Queen Victoria Street, London. Old linen, bandages, and dressings of all descriptions are urgently needed.

### Nursing in Switzerland.

### PRINCIPLES OF LA SOURCE.

By Dr. Charles Krafft, Director.

Ambroise Pare, the great French surgeon of the 16th century, said, speaking of one of his patients, "I dress his wounds, God heals him." This simple and modest speech, as far removed from the mysticism which prays without acting as from the rashness which acts without praying; this beautiful motto of the Christian physician; the care of the sick under the benediction, with the aid, of God;—this is the fundamental principle of La Source. To teach young women, married women, and widows compassion and care for those who suffer; to teach them to seek in the Gospel the light and strength indispensable to whoever wishes to live and serve as a child of God, such is the aim of the school. A magnificent aim; placed so high as to seem beyond reach, but attainable, through the grace of Him who never refuses His aid to those who believe in His goodness. A number of institutions designed to train religious nurses, place celibacy, renunciation of salary, the rule of obedience, and the wearing of a special garb at the foundation of their structure. To explain the origin of an organization of which the Holy Scriptures contain not a single trace, it must be remembered that our old Europe had long suffered the yoke of popes and priests; that certain Catholic errors still live in the minds of more than one Protestant. Thus it happens that the temptation of borrowing from Rome some of her convenient customs is very seductive. La Source, obeying other inspirations, follows a different path. For her nurses she desires liberty in Christ, spontaneity, the development of their capabilities, modesty in the exercise of their calling, in outward appearance nothing to attract either the curiosity or the admiration of the public. She wishes for them respect for the laws of God, who made woman the companion of man, who permits no other leader for our souls than Himself, and who dignifies all remuneration by declaring that the labourer is worthy of his hire. Upon her entrance into the school each pupil receives a short explanation of the principles to which the institution owes its origin, and which she is to follow and support. They are as follows:

### Principles.

The school called La Source aims to train capable and pious nurses for the sick. It differs in the five following points from other institutions which pursue an analogous course. (1) Its pupils conform to a common law only during their time of training. (2) It does not bestow the title "sister" on its pupils. (3) It does not require a special dress. (4) It does not exact celibacy. (5) The time of training finished, and the pupils having graduated as nurses, all money earned by them is received by them directly from their patients. The school placing at its foundation the five principles just enumerated, puts them in practice, that its example may show their justice.

### Explanation.

(1) Common Rule. During their training the pupils must submit, as they do in every school, to a common rule. But God, who gave each individual a conscience and a will, has by the words of the Saviour established for each one liberty of action. Our school, the time of training ended, does not impose any rule whatever on its pupils. Their individual liberty is thus entirely preserved. (2) Sisters. La Source does not give the title of Sister to its pupils or graduate nurses, believing that they have no more right than other Christian women who exercise a similar vocation to bear this special title. (3) Costume. La Source does not require its pupils or graduates to wear a special dress. They, being prepared for their mission, follow it in the world as do other Christians, protected by a husband, brothers, relations, friends, and by a strong evangelic education, and above all, by Him, Who directs and Who guards each of His children. (4) Celibacy. La Source does not consider celibacy indispensable to the functions of a hospital nurse. Among married women there have been, and there are to-day, many noble careers in the service of the sick, many examples of devotion. In virtue of this fact and of the higher law, the school admits to its teaching either married women, widows, or unmarried women. (5th) Salary. God said: "Thou shalt earn thy bread in the sweat of thy brow." The hospital nurse who, on principle, declines a salary, places herself above the common law. The nurse or sister, deprived of capital or of revenue, finds herself, if she leaves the institution after some years of service, deprived of the legitimate resources which her work ought to have assured to her. Let us add that the gratuity of this work is illusory. Since the support of sisters is assured during life to those who remain affiliated with the order, our school holds it honourable for women and for men alike to earn their daily bread. We see nothing in the law of God, either here or elsewhere, to be corrected. We render homage to those who, placed in a position of great wealth, consecrate it to the gratuitous care of the poor. But, if we see in this a privilege, we do not see in it a superiority. In our eyes, each nurse should remain free either to accept remuneration or to refuse it.

*(To be continued.)*

### Belgrave Hospital for Children.

### A DEMONSTRATION.

The demonstration for the Belgrave Children's Hospital took place on Sunday afternoon, October 29th, and was certainly a most impressive affair. Fortunately the weather was fine and the handsome banners showed to great advantage. There were three or four bands, a red Parcel Post van full of people, another of "Druids" in their pretty white dress, and a van load of little children with real lambs besides them; there was a hospital cot containing a child (not a patient), and various other attractive displays formed part of the long procession. The men wore the badges of different orders, such as Good Templars, Rechabites, &c. They marched around the neighbourhood from early morning, bands playing, money boxes rattling, some on long poles collecting from the spectators in the windows. Very tired did the poor men look as they passed for the last time on their way back from church, but many cheerfully smiled and doffed their hats to the spectators at the hospital windows.

One of the processionists seems very fond of visiting the little hospital, and every atom of the banner he carried had been made by his own hands, and he loves it more than anything else in the world. It is covered with mottoes on both sides, and is georgeous with red, white, and blue silk, waving ribbons and tassels. On the top of the pole is a splendid crown with a lion on the top, "Just the same as they have in the army," he says. The man is a railway porter, and works until two o'clock in the morning. He said he was up "very early" on that Sunday morning, "walking for the Hospital." "It's the forty-fifth time I've walked for hospitals with my banner. And do you know why I do it?" he said, "'Cos I was once very ill in the Gordon Hospital, and they was so kind to me; they cured me, you know."

"So you're grateful?"

"Grateful? that's it; and I'm the only man in the whole world that's not only been a total abstainer all his life, and a Good Templar, but has also made a flag by himself, and can't read nor write." There is something very attractive about the man who says all this, with frequent adoring glances cast at the banner. He has taken all the hospitals into his heart, "walking for them," because they "cured me, you know."

### Everybody's Opinion.

[Correspondence on all subjects is invited, but we cannot in any way be responsible for the opinions expressed by our correspondents. No communications can be entertained if the name and address of the correspondent is not given, or unless one side of the paper only be written on.]

### CHILDREN'S WARDS IN INFECTIOUS HOSPITALS.

Nurse Janet writes: I should be glad to know if any Hospital readers can tell me if there are children's wards at any free fever hospitals in England? The adult patients so often say on leaving that they have been quite comfortable, but they certainly found the noise of the children very trying at first. This matter is naturally chiefly important when the adults have a serious attack of fever, and are delirious or at any rate so weak as to require every advantage of environment. Most questions asked in The Hospital get satisfactorily answered, and doubtless mine will secure for me information from other and more experienced nurses.

### Nurses at Worthing.

(By a Resident.)

Private houses, homes of rest, and even chapels were used temporarily as hospitals during the recent epidemic of typhoid fever at Worthing, nurses being appointed to work at each establishment. Other houses were utilised for convalescents, and of the incessant and indefatigable workers and helpers enough good can never be said.

Probably only persons who lived in the town during those terrible months can adequately estimate the grievous distress and the universal sorrow. Although the epidemic may be looked upon as past, great misery and poverty remain. Not only the normal poor people, but lodging-house keepers and many others will suffer absolute want this winter.

Of the nurses who worked bravely and fearlessly, two are dead, but they one and all live in the hearts of their patients. Those who went from house to house, attending the sick in their own homes, were well-nigh worshipped by those to whom they ministered. "The nurse is a real lady! God bless her," said one enthusiastic sufferer, "she ain't afeard to soil 'er 'ands, she ain't; she tidied up the place when she'd done a-tidying me!"

One nurse in recording her experiences, told of a family of seven persons all stricken by the disease, and the wife died and the husband survived, a mere wreck of his former self, "and five sickly little bairns dependent on him for daily bread, which he also is too weak to earn."

Such tales are too numerous and sad to dwell on, and unhappily the relief fund is nearly exhausted. No wonder the approaching winter is dreaded by all classes. "Nothing but sorrow behind us, and only starvation before us," say sad-eyed women and anxious-looking men.

### Novelties for Nurses.

A good selection of patterns of warm and cheap materials have reached us from Hackett's Cardigan Works, High Street, Birmingham. The Swansdown Unshrinkable Flanelettes in all shades of colours are peculiarly attractive and suitable to this season. They are light, warm, and pretty, and certainly wonderfully cheap. A new dress material, double-width, stylish in pattern and low in price is called the Cheviette. We advise nurses requiring flannelettes to write for patterns to Mr. Hackett, who does not require his neat little sample packets returned, but says they "may be kept for future use."

### For Reading to the Sick.

### SHAPING THE STONES.

Most of us have been into a stonemason's workshop and seen stones of every shape and description lying about, some rough, just as they came from the quarry, others squared, smooth, and even, fit for the builder's hand. Now these latter did not become so of themselves, a great deal of time and labour had been spent upon them, and a great number of sharp tools had been used to bring them into proper order. We can all see the necessity and propriety of this, so we will study it as being a figure of God's dealings with us. St. Peter tells us that Christians are "living stones," built by God into the spiritual house of which Jesus Christ is "the chief corner-stone." But to make us "living stones" our Heavenly Father takes us out from the quarry, that is the world, and puts us into His workshop, the Church, from which He chooses those best fitted to be built into His spiritual temple. We therefore are naturally no more fit to be used in such a glorious work than are the stones just taken from the quarry ready for building; but we have been selected, cut out, severed, and brought away to be prepared. It is not for any goodness of our own, but the Father's free mercy and love through the Son, which has brought us into a state of salvation. God has chosen us, and as we are but clumsy, ill-shaped fragments, we must be wrought upon and prepared, we must be shaped and fashioned anew, our rough tempers polished off so that we may not disfigure the building.

Sometimes the Master Mason smites sharply with the hammer of anguish and pain, at others with the cold chisel of sorrow and affliction till our hard stony hearts take the form which God wills. Shall we blindly fret at these gracious dealings and wilfully choose rather to be cast on one side as unprofitable stones, than thus to be the subject of a chastening, purifying love? No, a thousand times no. Let the great Architect be busy with us, even though the work be painful and grievous, for the more we suffer, the fitter we shall be for a place of honour and usefulness in His temple. Which is the fairest stone in a beautiful church? That which has been longest under the carver's hand, that which has been patiently wrought on with many a sharp biting tool, hollowed out here, shaped away there, till it has become the crowning beauty of the whole. We may rest in hope, that we are being perfected in varied Christian graces by our sharp sufferings and stern chastisements, till we are the choicest work of the great Master Mason's hand. "Long and painful sickness," says a pious writer, "is often blest to the attainment of such patience and humility and meekness and thankfulness as is rarely won by those in health." Of such it may be said,

> "Many a blow and biting sculpture,
> Polished well those stones elect."

Then never murmur at the dealings of God, even if His hand presses heavily on us. We will rather say, "As Thou wilt, O my Father, only let my sickness be to Thy glory." If Jesus, "the chief corner-stone," was made perfect through suffering, the stone that is shaped and moulded by suffering is most fit to be placed near Him.

### Notes and Queries.

**Queries.**

(218) *Service.*—I want a place as attendant.—*S. G.*

(219) *Fever.*—Please tell me if a year's training in a fever hospital will fit me for a post as assistant nurse in a general hospital?—*Nurse Marian.*

**Answers.**

(218) *Service* (*S. G.*).—You had better advertise in The Hospital, stating what work you are qualified to undertake.

(219) *Fever* (*Nurse Marian*).—A year's training in fever nursing only will not fit you for holding the post you mention in a general hospital. You would be eligible for some infirmaries no doubt.

### Causerie from New England.

(By Our Boston Correspondent.)

A keen observer of human life has said, "Most men at heart are cowards." But heroism finds expression in phases of life where it is unrecognised until some striking instance forces our attention. Surgeons at the operating table, physicians in the wards for contagious diseases, nurses in the daily round of duty at the hospital, or amid the unsanitary conditions of district nursing, may not to the unthinking suggest anything heroic. But who that knows the fateful energy latent in the micro-organism can deny the heroic element that enters into the medical and nursing professions? Knowing well the danger, they yet gladly take the risk of personal peril for the sake of lessening the pains of humanity.

A late number of an American medical journal records the recent death of physicians from yellow fever, typhoid fever, diphtheria, and tuberculosis, resulting in each case from the exposure demanded by hospital work. It also calls attention to the serious illness of five officers in the Marine Hospital Service, caused by infection from the performance of their medical duties. Surely self-poise and the supreme gift of self-sacrifice can alone be sufficient for these things?

The heroic element, which makes the substratum of a physician's character, was brought afresh to mind by the memorial service for the late Dr. Walter Gay Stebbins, held at the Boston City Hospital, on Sunday afternoon. This young man, of rare attainments and unimpeachable character, had finished his term as house physician and surgeon, and returned to the hospital for a few weeks to assist in the admission of patients during the absence of one of the staff. In examining a child with malignant diphtheria, he caught the infection, which proved fatal. This service is worthy of attention, not for the exquisite singing or tributes of flowers, not for the notable gathering of the medical staff, nor the young doctors, nor the white-capped nurses, nor even for the admirable, sympathetic analysis of the character of Dr. Stebbins, given by his co-worker, Dr. Taylor, but for the strong words of sound, practical wisdom offered by Dr. David W. Cheever, senior surgeon to the hospital, and one of the leading American practitioners. I have his kind permission to give them in full, and every hospital worker should ponder them well.

"As my house surgeon, Dr. Stebbins was intelligent, courteous, faithful, kind, self-controlled, I felt easy in leaving the hospital, because confident that my representative there was competent, and always at his post. Few realize how much the success of operations depends upon the care afterwards, and none but the visiting surgeon appreciates the full value of a reliable assistant. These qualities of Dr. Stebbins, had he lived, would have ensured his success. It seems to us that he died prematurely, and that his death cut short an unfinished life. But is there any life, or anything in this world, that is finished? We are all removed from this life, some early and some late, but always with a sense of incompleteness. A beautiful world, which we never know and never learn, is all around us; and we are snatched away from it before we recognize or appreciate its full meaning. This is life—it was so meant, it is so planned. We cannot alter it, we cannot decide upon the order of our going. What then can render this incompleteness more complete to us? What can reconcile us to this half-life? Solely, I conceive, the consciousness that we learn all we can; that we do all we can; that we develop ourselves all we can. This is to be mature at any age and to be old in youth. This our friend did, and to the fullest extent; he improved his opportunities; he worked to the extent of his strength; he lived all he could; he developed all he could: he was complete for his years; he was matured and not young. Whenever such a person dies, his life is a success, for him a satisfaction, if for us a regret. A life ended in the service of sick fellow-beings, is a noble conclusion. It consecrates its memory. It satisfies both expectation and retrospect. The lesson of such a life and death to us should be uplifting, encouraging, but never depressing. We aspire, we imitate, we follow, satisfied if we do as well. It may not be inappropriate, also, to consider the practical lesson to ourselves of such a life. The doctor, the medical student, the nurse are fearless of contagion, of inoculation. They should be fearless. Fear is the ally of death and disease. Timidity invites contagion, depresses vitality, encourages sickness. Fear not, but also be prudent. Caution, care, are duties as much as courage. I would urge on the hospital student, attendant, and especially on the nurse, to take every known precaution against infection. Nature has endowed us with an armour against disease. That armour is high or perfect health, vital resistance, which sheds germs like water, cleanliness most scrupulous, exercise, fresh air, sunlight, a clear conscience, these are the means to ward off sickness. Never allow yourself to eat until you have freshly washed your hands and mouths. Take care of contagious cases fearlessly, but do not nurse and coddle them in needless proximity. Disinfect, cleanse, watch the soundness of your hands, neglect no abrasion of the skin, gargle with disinfectants freely, bathe often, sleep, eat, go out of doors. This is our duty as much as the care of others. Do justice to the sick and do justice to yourself. The longer you live the more good you can do, and this should urge you to prolong your lives to the utmost that you can. And when the end comes may we go assured that we have done the best we knew how; have lived up to the light we had; have little to be remorseful for. If so, we, like our departed friend, shall be old, though young, satisfied in conscience, an honour and an example to the world."

The Training School for Nurses was also represented in this service by one of the pupils, who read that most appropriate poem of Whittier's "The Eternal Goodness." Dr. Rowe added a few words, in the course of which he said: "My experience as Medical Superintendent of this hospital has seen nearly two hundred and fifty young men tested by the stress of hospital duties, and I have followed their after career with justifiable pride. No severer ordeal can be sustained by a young man than the crucial term as house physician and surgeon in a large municipal hospital. What a man gains, he gains by sheer force of character. Under these trying conditions Dr. Stebbins proved himself 'a knight without fear and without reproach.'"

"Without reproach," is the verdict of those associated with him in the routine of daily life where weakness or selfishness cannot be hidden. "Without fear," also, for is not the heroic an element in the life of a physician, who must stand by the bedside of loathsome or malignant or infectious disease? Our loving homage goes out spontaneously to

One who never turned his back, but marched breast forward,
Never doubted clouds would break,
Never dreamed, though right were worsted, wrong would triumph,
Held we fall to rise, are baffled to fight better, sleep to wake.

Such a service does more than honour the virtues of the dead. It has a distinct influence for moral elevation throughout the whole corps of hospital workers. It cannot fail to impress the young men that character is the only thing after all; that "no one can do much for others who is not much himself." This service was also a distinct recognition of the difficulties and dangers that render the daily life of doctor and nurse hazardous, and therefore heroic. Nothing is a stronger incentive to excellence than a sense that it is sure to be appreciated.

This aspect of heroism in medicine and nursing puts to shame the young woman who said: "I'm not going to apply for admission to the Training School at —— Hospital. I'd rather go to one without contagious wards—there's less risk." Evidently she is not "the stuff of which martyrs are made." Carlyle did not go astray in tracing the etymology of "valour" and "value" to the same parent stock.

O. M. E. R.

### The Book World for Women and Nurses.

[We invite Correspondence, Criticism, Enquiries, and Notes on Books likely to interest Women and Nurses. Address, Editor, The Hospital (Nurses' Book World), 428, Strand, W.C.]

Woman's Mission. Papers on the Philanthropic Work of Women. Edited by the Baroness Burdett-Coutts. (Sampson Low, Marston, and Co.)

Miss Nightingale's paper on "Sick Nursing and Health Nursing," contributed to the collection of papers on Women's Work, edited by the Baroness Burdett-Coutts, for the Women's Section of the Chicago Exhibition, will be read with respect by all nurses, not only on account of its aged writer's claim to a hearing, but also on account of the strong common-sense of many of her *dicta*. She complains, and justly, that though the world depends on women to bring children into the world and to rear them to healthy man or womanhood, no attempt is made to teach women the very simplest laws of hygiene, unless they elect to be doctors or nurses, and are trained accordingly. Miss Nightingale says, "It is the want of 'the Art of Health,' which has only lately been discovered." People will go to any trouble and expense to get the best doctors and nurses when they fall ill, but they do not contrive to gain instruction for themselves or their children (the future fathers and mothers of our race) as to how sickness may be in great measure avoided. How much of it might be avoided is best known to doctors and and nurses. Intelligent attention to small things—cleanliness, prevention of chills, &c., &c.—may make a delicate child into a healthy one, or a naturally healthy child may become a chronically diseased one through neglect of most ordinary precautions, even in households where the children are idolized. Miss Nightingale shows all through her paper how highly she rates the nurse's responsibility, which is, in fact, a sacred trust. To our thinking, as the young curate stands in relation to his rector so almost stands the nurse to the doctor—they have all as it were "taken orders"—the clergyman to minister to the soul's health, and the doctor and the nurses, his aide-de-camps, to minister to the body. How entirely we have to trust our dear ones to the trained nurse whom the doctor orders us to procure. If she be not one with the very loftiest views as to the terrible responsibility she takes on herself then were it far better for her as well as her patients that she should cease to call herself a nurse. We are led to speak strongly, for in these days so many dissatisfied girls think to kill *ennui*, and, under protection of the nurse's uniform, to lead a life of independence, excitement, and amusement. The majority of nurses are noble-hearted and devoted women. Whether they be "born ladies" or not their hearts are so in their work that those not so born become, through sympathy with suffering, refined enough to attend the highest in the land without jarring on their susceptibilities; while those who are so born find their early training in good manners and the ways of cultivated society a help to them in dealing with their patients from the first, thereby affording a slight compensation for the certain disadvantages which inexperience in domestic work hampers them with during their novitiate. Miss Nightingale urges for nurses first the most complete and thorough training; she says, "the physician prescribes for supplying the vital force, but the nurse supplies it. Training has to make her, not servile,

THE BOOK WORLD FOR WOMEN AND NURSES—*continued.*

but loyal to medical orders. True loyalty to orders cannot be without the independent sense or energy of responsibility which alone secures real trustworthiness. Training is to teach the nurse how to handle the agencies within our control, which restore health and life, in strict intelligent obedience to the physician's or surgeon's power or knowledge" Again, she asks, "What is discipline? It is the essence of moral training, all that goes to the full development of our faculties, moral, physical, and spiritual, not only for this life, but looking on this life as the training ground for the future and higher life. Discipline embraces order, method; and as we gain some knowledge of the laws of Nature ('God's laws') we not only see order, method, a place for everything, to each animate or inanimate thing or being its own appointed work, but also we find no hurry, we learn to have patience with our circumstances and ourselves, and we learn to become more diciplined, more content to work where we are placed, more anxious to fulfil our appointed work than to see the result thereof."

### CURRENT PERIODICALS.

The Leisure Hour for November can be fairly complimented on having catered for all tastes. It contains a wonderfully large amount of knowledge and amusement. With the number commences a new story, entitled "Farm and Town," by the author whom "Helen's Babies" has made very popular. Besides the popularity of the author, "Farm and Town" has this to recommend it, that a really reasonable portion of the story is presented to its readers, which is a rare occurrence in the serials in monthly periodicals with a wide range of subjects of the more solid form of literature. This month the *Leisure Hour* contains excellent short paragraphs on criticism, under the heading of "Second Thoughts on Books." A description of Professor Bell's interesting invention, "The Radiophone," makes the science pages especially interesting, and "Thought Splinters" contains some excellently terse truisms.

In the Cornhill Magazine, "With Edged Tools," the story whose authorship remains unrevealed, continues its interest. The characters, if not all pleasant ones, are strongly drawn, and the situations in which they are placed serve to bring them out in bold relief. Of the shorter stories the character note, "The Caretaker," is perhaps the most interesting. Many a frequenter of city offices and chambers will recognise an old friend, for whom, in spite of frequent delinquencies, they retain a kindly feeling. "In Summer Heat" and "January Days in Ceylon" bear a nearer affinity than their geographical standpoints would suggest.

## Figures and Tables

**Figure f1:**